# Can Tracking Data Help in Assessing Interpersonal Contact Exposure in Team Sports during the COVID-19 Pandemic?

**DOI:** 10.3390/s20216163

**Published:** 2020-10-29

**Authors:** Bruno Gonçalves, Romeu Mendes, Hugo Folgado, Pedro Figueiredo, Bruno Travassos, Henrique Barros, Adalberto Campos-Fernandes, Paulo Beckert, João Brito

**Affiliations:** 1 Departamento de Desporto e Saúde, Escola de Ciências e Tecnologia, Universidade de Évora, 7000-645 Évora, Portugal; hfolgado@uevora.pt; 2 Comprehensive Health Research Centre (CHRC), Universidade de Évora, 7000-645 Évora, Portugal; 3 Portugal Football School, Portuguese Football Federation, 1495-433 Oeiras, Portugal; romeu.mendes@fpf.pt (R.M.); pedro.figueiredo@fpf.pt (P.F.); bfrt@ubi.pt (B.T.); paulo.beckert@fpf.pt (P.B.); joao.brito@fpf.pt (J.B.); 4 EPIUnit—Instituto de Saúde Pública, Universidade do Porto, 4050-600 Porto, Portugal; henrique.barros@ispup.up.pt; 5 Research Center in Sports Sciences, Health Sciences and Human Development (CIDESD), University Institute of Maia (ISMAI), 4475-690 Maia, Portugal; 6 Research Center in Sports Sciences, Health Sciences and Human Development (CIDESD), University of Beira Interior, 6201-001 Covilhã, Portugal; 7 Escola Nacional de Saúde Pública, Universidade Nova de Lisboa, 1600-560 Lisboa, Portugal; adalberto.fernandes@ensp.unl.pt

**Keywords:** positional data, tracking systems, football, SARS-CoV-2, pandemic, physical distance

## Abstract

During the COVID-19 pandemic, the temporary closure of physical activity and sports facilities, and the generalized cancellation or postponement of sports events have a massive impact on social and economic development. In this study, we explored the feasibility of using tracking data from a football match to assess interpersonal contact between individuals by calculating two measures of respiratory exposure. The dynamic tracking positioning of all players and referees during one international football match was analyzed. For each individual, two measures of respiratory exposure were calculated, based on the 2 m interpersonal distance recommendations for contact tracing for COVID-19 control. Overall, individuals spent a median of 0:12 mm:ss (IQR = 0:45 mm:ss) exposed to interpersonal contact of fewer than 2 m from others. The highest value of exposure was observed between two players of opposing teams (6:35 mm:ss). The results suggest that tracking data can be used to assess respiratory exposure to interpersonal contact in team sports, such as football. The measures of exposure calculated can be used to the prompt identification of high-risk contacts of COVID-19 cases during a match or a training session, but also the risk stratification of different sports and physical activities.

## 1. Introduction

The COVID-19 epidemic disrupted the world of physical activity and sports. Global social and physical distancing measures resulted in the closure of sports clubs, gyms and health clubs, stadiums, swimming pools, dancing and fitness studios, parks and playgrounds, and people were generally not allowed to engage in their regular individual or group sporting or physical activities outside of their homes [1].

Under social confinement conditions, most individuals tend to be less physically active, have more extended periods in sedentary behaviors, irregular sleep patterns, and worse diets, which may result in body weight gain and loss of physical fitness [1,2]. Though physical activity is an essential determinant of health, and physical inactivity and sedentary behavior are major risk factors for non-communicable diseases [3], which are associated with more severe COVID-19 outcomes, including death [4]. For this purpose, proper management of noncommunicable diseases and their respective risk factors is vital to optimize public health outcomes and reduce the impact of the COVID-19 pandemic on individuals and society [5].

On the other side, the temporary closure of physical activity and sports facilities, and the generalized cancellation or postponement of sports events at international, regional, and national levels had a massive impact on social and economic development. Many millions of jobs are at risk globally, not only for sports professionals but also for those in related retail and sporting services industries connected to leagues and events [1].

Football is the sport with the highest number of athletes involved worldwide. Besides its impact on the global economy, football practice, even at the recreational level, has significant health benefits in individuals with non-communicable diseases [6,7]. However, resuming sports and football after social confinement might present several COVID-19 risks that must be controlled, especially exposure to interpersonal contact during play [8,9].

Physical distancing is one of the most important measures to prevent SARS-CoV-2 transmission mainly through respiratory droplets [10,11]. However, physical distancing is not possible in many team sports, including football. Actually, the World Health Organization (WHO) considered contact sports as COVID-19 high-risk sports, due to physical and close contacts among players [12].

In the current study, we explored the feasibility of using tracking data from a football match to assess interpersonal contact between individuals. For this purpose, we calculated and analyzed two measures of respiratory exposure during a football match.

## 2. Materials and Methods

### 2.1. Participants and Equipment

This cross-sectional exploratory study analyzed one international football match from elite adult male players, previously recorded in 2019. The final score of the match was 3-3. Dynamic tracking positioning of all players from both competing teams (i.e., 22 players and six substitutes) and referees (one referee and the two assistant referees) was captured and transformed into two-dimensional coordinates using the TRACAB Optical Image Tracking System at 25 Hz (https://chyronhego.com). The system uses super-HD cameras and patented image processing technology to deliver live tracking of all moving objects with a maximum delay of just three frames (https://chyronhego.com/) and it is used in several professional soccer leagues including English Premier League, German Bundesliga and Spanish La Liga Dutch Eredivisie, Danish Superliga, as well as European matches in the UEFA Champions’ League and International matches in UEFA and FIFA tournaments. There have been many scientific studies based on TRACAB’s data, dealing with the attacking performance in football [13], individual ball possession [14], spatial-temporal features that describe a team match demands when considering the effects of the quality of opposition in elite football [15]. It has been previously validated where it was compared against simultaneously recorded measures of a reference system (VICON motion capture system) [16]. The root means square error was 0.09 m in position measurements, 0.09 m⋅s^−1^ in instantaneous speed and 0.26 m⋅s^−2^ in accelerations. For total distance travelled and peak speed, trivial deviations were identified compared to the reference (0.42 ± 0.60% and <0.5%, respectively). For the current investigation, sampling frequency was set at 5 Hz to match the frequency of a typical football sports tracker (e.g., global position system) and to reduce the computing time.

Overall, 31 individuals and 930 different possible pairing relations were considered into analysis for every point in time throughout the match according to their football team (A and B) and position (Goalkeeper [GK], Left Defender [LD], Central Defender [CD], Right Defender [RD], Left Midfielder [LM], Central Midfielder [CM], Right Midfielder [RM], Forward [W]), or refereeing role (Referee [REF], Assistant Referee [AREF])—e.g., A:GK vs. A:LD; A:GK vs. B:CD, A:GK vs. REF, etc.

The study protocol was approved by a university ethics committee (CE-UBI-Pj-2020-043).

### 2.2. Procedures and Data Processing

Two measures of respiratory exposure were calculated for each individual. These measures were based on the 2-m interpersonal distance from the European Centre for Disease Prevention and Control (ECDC) recommendations for contact tracing for COVID-19 control [14] and adapted from the work of Knudsen et al. [17].

#### 2.2.1. Measure of Exposure 1

For each individual, exposure to interpersonal contact was calculated considering the accumulated time positioned for less than 2 m from the other individuals during the match.

#### 2.2.2. Measure of Exposure 2

Since respiratory droplets can be left in the air by moving individuals [18,19], the second measure of exposure was calculated adding to the previous measure of exposure (Measure of Exposure 1), the weighted time that each individual was exposed to the tracks of respiratory droplets left by the movements of the other individuals (also considering the 2 m distance), accordingly to the timeframe of each path.

**Figure 1:**
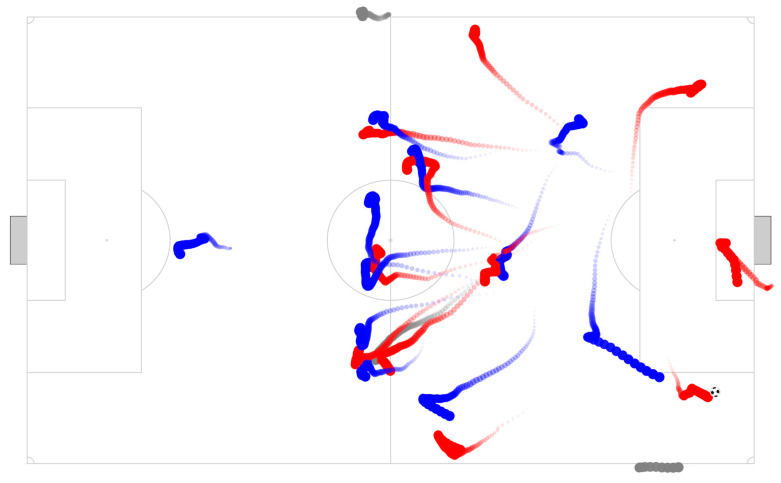
Match frame animation. Teams are colored with blue and red, and referees in grey. Each track shows a delay position of 15 s, which represents 75 data points (15 s * 5 Hz). For each data point, an exposure score was attributed according to the time position.

**Figure 2:**
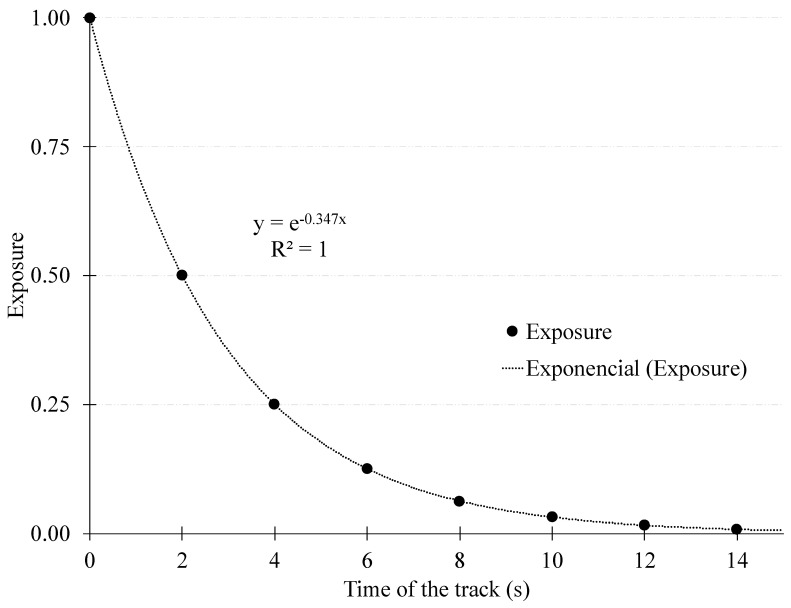
Exponential function calculating the exposure score of the contact with the individuals and their tracks of respiratory droplets. The exposure declines to a half every 2 s. The window for calculation was set between 0 and 15 s, where the exposure score is 0.005. Adapted from Knudsen et al. [17].

Given the droplets size distribution [20,21] and the evaporation-falling curve [21], a timeframe of 15 s was considered to create a track of an individual’s previous movement (Figure 1). Within each track, exposure exponentially declined at a rate of 50% every 2 s (Figure 2), modeled by the equation: Exposure score = e^−0.347 * time of the track (s).

For example: if an individual A and an individual B were less than 2 m apart, the exposure score was 1 for both individuals; if the individual A was positioned within 2 m where the individual B was positioned 2 s before, the exposure score was 0.5—but only for individual A.

The weighted time of exposure to interpersonal contact was then calculated as the sum of all exposure scores at any given time, regarding all the possible pairings of individuals, divided by the positional data sampling frequency.

All calculations were processed in Matlab^®^ (The MathWorks Inc., Natick, MA, USA). Results are presented in median and interquartile range (IQR) and in mean and coefficient of variation (CV).

## 3. Results

### 3.1. Measure of Exposure 1

**Figure 3:**
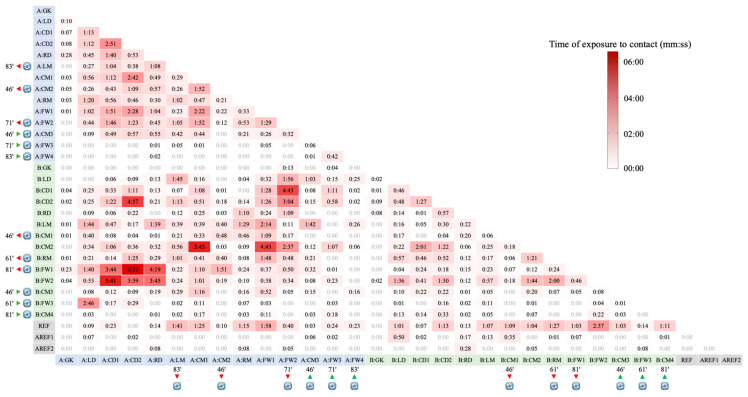
Accumulated time (mm:ss) each individual was positioned for less than 2 m from other individuals (players and referees). Blue cells represent players from team A, green from team B, and grey the referees. The symbol depicts the players that were substituted and the match time of occurrence. GK = Goalkeeper, LD = Left Defender, CD = Central Defender, RD = Right Defender, LM = Left Midfielder, CM = Central Midfielder, RM = Right Midfielder, FW = Forward, REF = Referee, AREF = Assistant Referee.

Figure 3 presents the accumulated time each individual (player or referee) was positioned for less than 2 m from other individuals during the match. Overall, individuals spent a median of 0:12 mm:ss (IQR = 0:45 mm:ss) exposed to interpersonal contact of fewer than 2 m from others. The highest value of exposure was observed between players A:CD2 and B:FW1 (6:35 mm:ss). The average exposure time per pair of individuals was 0:32 mm:ss (CV = 165%).

### 3.2. Measure of Exposure 2

**Figure 4:**
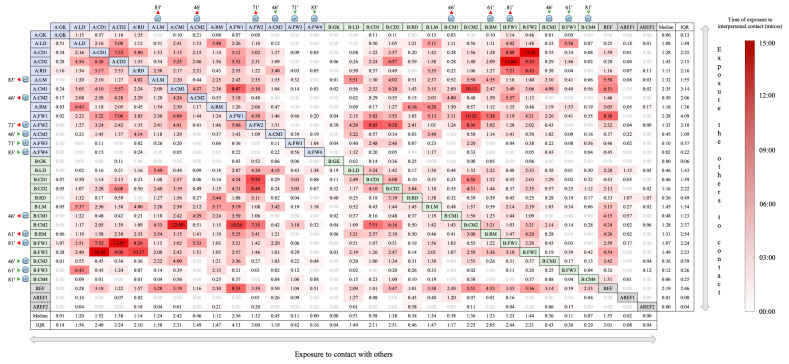
Accumulated weighted-time (mm:ss) each individual was exposed for less than 2 m to other individuals (players and referees) and their tracks of respiratory droplets left by their movements. Blue cells represent players from team A, green from team B, and grey the referees. The symbol depicts the players that were substituted and the match time of occurrence. The figure allows double information and should be read according to rows and columns: rows show the exposure to contact with other individuals (e.g., A:GK was exposed during 1:15 mm:ss to A:LD, 0:57 mm:ss to A:CD1, etc.; columns show the time that each individual exposed the others to contact with himself (e.g., A:GK exposed A:LD to 0:51 mm:ss, A:CD1 to 0:34 mm:ss, etc.). The last rows and columns show the corresponding median and interquartile range (IQR). Darker shades indicate a higher time of exposure to interpersonal contact. GK = Goalkeeper, LD = Left Defender, CD = Central Defender, RD = Right Defender, LM = Left Midfielder, CM = Central Midfielder, RM = Right Midfielder, FW = Forward, REF = Referee, AREF = Assistant Referee.

Figure 4 shows the time each individual was positioned for less than 2 m from other individuals plus the time of exposure to the tracks of respiratory droplets left by the movements of other individuals (also considering the 2 m distance). Overall, each individual was exposed to 00:44 mm:ss (IQR = 02:04 mm:ss). The highest value of exposure was observed between players A:CD2 and B:FW1 (14:10 mm:ss). The average per pair of individuals was 01:31 mm:ss (CV = 139%).

Figure 4 allows double information since the time of exposure to interpersonal contact is not symmetric between pairs of individuals. For example, B:FW2 and A:CD1 revealed the highest time of exposure during the entire match between players (14:10 mm:ss), which represents the time that B:FW2 was exposed to A:CD1 (see row interception B:FW2 with column A:CD1). On the other side, A:CD1 was exposed to B:FW2 during 13:26 mm:ss (see column interception A:CD1 with row B:FW2). As the interpersonal contact measured within the 2 m distance is mutual, the higher value for player B:FW2 represented more time spent near the track of respiratory droplets of player A:CD1.

For referees, the time of exposure was well distributed among both teams. While AREF1 and AREF2 presented low values of time of exposure to interpersonal contact, the REF presented higher values (both being exposed and exposing) mainly in interaction with A:FW1 (8:33 mm:ss and 8:36 mm:ss, respectively). Moreover, the REF presented a median of 2:19 mm:ss (IQR = 2:46 mm:ss) of exposure to contact with others, and 1:55 mm:ss (IQR = 3:01 mm:ss) in exposing the others to contact with himself.

## 4. Discussion

The results suggest that tracking data can be used to assess respiratory exposure to interpersonal contact in team sports, such as football.

To identify and manage the contacts of suspected or confirmed COVID-19 cases, the ECDC defined high-risk exposure to interpersonal contact as having had face-to-face contact with a COVID-19 case within 2 m for more than 15 min; low-risk exposure was defined as having had face-to-face contact with a COVID-19 case within 2 m for less than 15 min [22].

Though, using the ECDC recommendations mentioned above, and based on the Measure of Exposure 1 (Figure 3), if player A:CD2 was considered a suspected or confirmed COVID-19 case in the 48 h after the match, the current tracking analysis revealed that: no contact within 2 m for more than 15 min was observed with other player or referee; contacted within 2 m for less than 15 min was observed between 23 players and one assistant referee (AREF1), with the maximum contact time observed with player B:FW1 (6:35 mm:ss); and no contact within 2 m was observed between four players (B:CM4, B:GK, A:FW3 e A:FW4), the referee (REF) and one assistant referee (AREF2).

Using the same example (i.e., player A:CD2), but with the Measure of Exposure 2 (Figure 4, columns data; that includes a contact to tracks of respiratory droplets besides interpersonal contact), the match analysis revealed that: no contact within 2 m for more than 15 min was observed with other player or referee; contacts within 2 m for less than 15 min was observed between 25 players with the referee (REF) and one assistant referee (AREF1), with the maximum contact time observed with player B:FW1 (13:05 mm:ss), and the contact with 11 players and AREF1 was less than 02:00 mm:ss; and no contact within 2 m was observed between four players (A:FW3, A:FW4 and B:CM4) and one assistant referee (AREF2).

The results obtained for this football match cannot be extended to football training sessions and other activities involving football teams. In addition, the results cannot be extrapolated to non-elite football players and referees, where playing intensity, technical skills, and tactical positioning can differ. However, this model of analysis can be performed for other football matches or training sessions, and even for other sports and physical activities in order to support the risk stratification of interpersonal contact exposure.

We used the risk exposure distance*time recommendations from the ECDC [22] just as an example to show the applicability of the current analysis. Though, calculations can be performed with other interpersonal distances and adjusted to the need to analyze more or fewer proximity levels between individuals during sports practice and the respective interpersonal contact analysis.

It should be noted that the definition of distance cut-offs for physical distancing for the prevention of COVID-19 transmission is a very debatable issue. The WHO recommended a minimum distance of at least 1 m between people to limit the risk of interpersonal transmission, both for social distancing [11] and sports competitors (when applicable) [12]. This recommendation was based on a systematic review and meta-analysis of observational studies (both health-care and community settings) that revealed a reduction in risk of 82% with a physical distance of 1 m (adjusted OR 0.18, 95% CI [0.09, 0.38]), and that every additional 1 m of separation more than doubled the relative protection (data available up to 3 m) [23,24].

This way, several researchers have been studying the safe distance for different physical activities practice, including walking, running, and football [17,25,26]. The study from Knudsen et al. [17] analyzed 14 elite football matches using a model with a 1.5-m distance to a supposed infected player and the track of respiratory droplets. On average, other players were positioned within the risk zone of the infected player for 01:28 mm:ss. We believe the model used in the current investigation may offer useful information, since we calculated and presented the time of exposure to interpersonal contact for every possible pair of individuals during the match, allowing to identify different levels of exposure between individuals, including the contacts with a suspected or confirmed COVID-19 case. A visual inspection to Figure 4 highlights a diagonal color trend (bottom left to top right), revealing that players were more exposed to, and exposed more, opponents rather than teammates.

However, caution should be taken on the current lack of information about transmission and risk models, specifically regarding SARS-CoV-2 [10,27]. In the future, the current approach may be adapted to forthcoming evidence and models. Digital technology should be integrated into policy and response of the COVID-19 pandemic, facilitating planning, surveillance, and contact tracing [28]. Though, tracking systems can be used to analyze sports and physical activities and evaluate different levels of risk exposure, allowing the definition of a progressive resuming to sports after lockdown periods.

## 5. Conclusions

Tracking data can be used to assess respiratory exposure to interpersonal contact in sports and physical activities.

The measures of exposure calculated and analyzed in this study can be used for the prompt identification of high-risk contacts of a suspected or confirmed case of COVID-19 during a match or a training session, and thus, to intervene and interrupt further onward SARS-CoV-2 transmission.

This model of analysis, based on digital technology, can also have an essential role in the risk stratification of different sports and physical activities in the scope of COVID-19 management.
